# Ontogeny-Driven *rDNA* Rearrangement, Methylation, and Transcription, and Paternal Influence

**DOI:** 10.1371/journal.pone.0022266

**Published:** 2011-07-12

**Authors:** Yih-Horng Shiao, Robert M. Leighty, Cuiju Wang, Xin Ge, Erik B. Crawford, Joshua M. Spurrier, Sean D. McCann, Janet R. Fields, Laura Fornwald, Lisa Riffle, Craig Driver, Octavio A. Quiñones, Ralph E. Wilson, Kazimierz S. Kasprzak, Gregory S. Travlos, W. Gregory Alvord, Lucy M. Anderson

**Affiliations:** 1 Laboratory of Comparative Carcinogenesis, National Cancer Institute at Frederick, Frederick, Maryland, United States of America; 2 Data Management Services Incorporated, Frederick, Maryland, United States of America; 3 Science Applications International Corporation-Frederick, Frederick, Maryland, United States of America; 4 National Toxicology Program, Research Triangle Park, North Carolina, United States of America; Tulane University Health Sciences Center, United States of America

## Abstract

Gene rearrangement occurs during development in some cell types and this genome dynamics is modulated by intrinsic and extrinsic factors, including growth stimulants and nutrients. This raises a possibility that such structural change in the genome and its subsequent epigenetic modifications may also take place during mammalian ontogeny, a process undergoing finely orchestrated cell division and differentiation. We tested this hypothesis by comparing single nucleotide polymorphism-defined haplotype frequencies and DNA methylation of the *rDNA* multicopy gene between two mouse ontogenic stages and among three adult tissues of individual mice. Possible influences to the genetic and epigenetic dynamics by paternal exposures were also examined for Cr(III) and acid saline extrinsic factors. Variables derived from litters, individuals, and duplicate assays in large mouse populations were examined using linear mixed-effects model. We report here that active *rDNA* rearrangement, represented by changes of haplotype frequencies, arises during ontogenic progression from day 8 embryos to 6-week adult mice as well as in different tissue lineages and is modifiable by paternal exposures. The *rDNA* methylation levels were also altered in concordance with this ontogenic progression and were associated with *rDNA* haplotypes. Sperm showed highest level of methylation, followed by lungs and livers, and preferentially selected haplotypes that are positively associated with methylation. Livers, maintaining lower levels of *rDNA* methylation compared with lungs, expressed more *rRNA* transcript. *In vitro* transcription demonstrated haplotype-dependent *rRNA* expression. Thus, the genome is also dynamic during mammalian ontogeny and its rearrangement may trigger epigenetic changes and subsequent transcriptional controls, that are further influenced by paternal exposures.

## Introduction

Gene rearrangement is required for development in selected cell types, such as mating-type switch in yeasts, diversification of variant surface glycoprotein in trypanosomes, amplifications of chorion protein genes for eggshell production in *Drosophila*, and maturation of B lymphocytes in humans [1]. Environmental stresses are capable of modulating these events and many of the programmed gene rearrangements, for examples, inversion control of flagellar protein in *Salmonella* and deletion/inversion of V(D)J immunoglobulin gene in mammals, are known to alter gene transcription [2]. Environmental stress responses that are transmissible to following generation(s) via genetic and epigenetic mechanisms have been detected in many organisms, including bacteria, fungi, plants, invertebrates, and mammals [3]–[5]. The *rDNA* gene, tandemly repeated in multiple chromosomes in many unicellular and multicellular organisms [6], is a hotspot area for homologous recombination [7], [8]. Amplification of the *rDNA* repeats is observed during oogenesis in *Xenopus* and macronuclear development in *Tetrahymena* [1]. Direct allelic reduction of the *rDNA* repeats in *Drosophila* lessened heterochromatin formation and gene silencing in unlinked genes elsewhere in the genome [9], suggesting a fundamental role of the *rDNA* structure change and its transcriptional regulation in organismal development. It is unclear whether the same *rDNA* rearrangement-mediated genomic controls also operate in mammalian ontogeny and how paternal exposures shape the processes.

In this study, we examined the roles of the *rDNA* genetic and epigenetic dynamics in mouse ontogeny and effects of two paternal stressors, namely, intraperitoneal injection of Cr(III) or acidic saline at a postmeiotic male germ-cell stage. The paternal treatment was to test the hypothesis that father experience may be transmissible to offspring. Furthermore, these genetic and epigenetic information may offer a clue to our previous findings of increased tumor incidence and changes in expression profile in the offspring following paternal treatment with Cr(III), compared with acid saline as a control [10], [11]. Cr(III) is known to reduce blood glucose [12]. Acidic saline is considered here as an independent stressor because it mimics peritoneal acidosis [13]. Putative structural rearrangements of the *rDNA* multiple-copy gene were determined by genotyping of haplotypes [14]. Since *rDNA* structural alteration is able to remodel epigenetic marks [9], the *rDNA* methylation was also quantified concurrently to determine epigenotype established along with ontogenic DNA replication and cell division. If *rDNA* rearrangement occurs, the haplotype frequency and epigenotype are likely to be varied throughout ontogeny. The levels of the *47S* pre-*rRNA*, the precursor of *rRNA* biogenesis, were further measured in adult tissues as a functional output of *rDNA* genotype and epigenotype.

## Results and Discussion

### Changes of *rDNA* haplotype frequency and methylation during ontogeny

**Figure 1:**
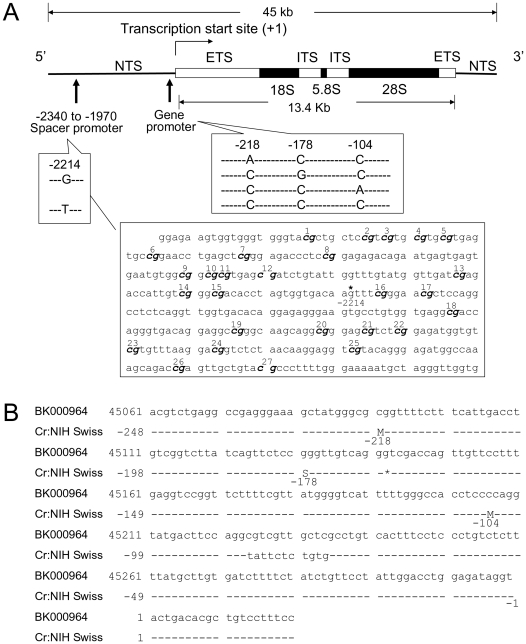
The mouse *rDNA* gene. (A) Locations of CpG sites and G/T SNP in a spacer promoter region (−2340 to −1946) and 4 sequence variants (haplotypes) with unique SNP combinations in the gene promoter. *G/T SNP; NTS: nontranscribed spacer; ETS: external transcribed spacer; ITS: internal transcribed spacer. (B) Sequence alignment of a Cr:NIH Swiss *rDNA* gene promoter region, upstream of the transcription start site 1, to the GenBank BK000964. The Cr:NIH Swiss sequence was obtained by cloning and dideoxy sequencing (M: A/C SNP; S: C/G SNP; *deletion).

Pyrosequencing-based quantitative assays with a manufacturer-determined resolution of less than 5% were used to quantify single nucleotide polymorphism (SNP) frequencies and DNA methylation in the *rDNA* repeats in this large study of thousands of samples. This sequencing-by-synthesis technique provides an advantage to detect small population of sequence variant directly from amplified DNA segments [15]. The standard deviation for duplicate analyses here ranged from 1.25% to 3.97%. In addition to the G/T polymorphism in the spacer promoter, the relative frequencies of four sequence variants or haplotypes, defined by three SNPs in the primary gene promoter that were identified in the current study (Figure 1), were measured to detect *rDNA* rearrangement. Variants of ACC, CGC, CCA, and CCC sequences (GenBank accession JF262063, JF262064, JF262065, and JF262066, respectively) were detected with a combined frequency of about 99% out of over 160 DNA clones from 19 mouse sperm samples. Predominant and consistent presences of ACC, CGC, CCA, and CCC among 8 hypothetical haplotypes from 2 SNPs each at 3 sites support unequal homologous recombination, a known major recombination event for *rDNA* repeats [7], not gene conversion. For epigenotype, the percentages of DNA methylation at CpG 19 to 23 sites, representative of a total 27 CpG dinucleotides in a region of the spacer promoter (Figure 1A, see also Materials and Methods) were quantified in the same DNA samples for SNP genotyping.

**Figure 2:**
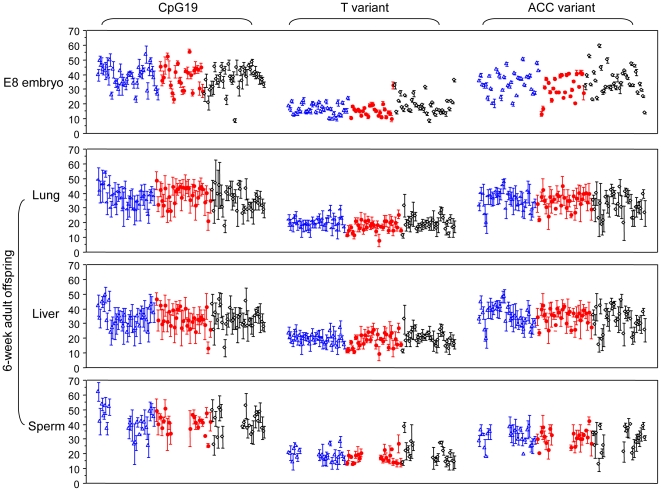
Increase of littermate-to-littermate variation during ontogeny. Within-litter variation, represented by error bars, of *rDNA* haplotype frequencies (%, T and ACC) and CpG19 methylation (%) widened from E8 embryos to 6-week adult tissues in 3 paternal treatment lineages (genders combined; blue: acidic saline; red: Cr; black: untreated). The average litter size is 9 at E8 embryo and 8 at 6-week adult stage. The plots are displayed as mean±1x standard deviation to illustrate the degree of variation.

To test the hypothesis that *rDNA* gene rearrangements and methylation changes occur during ontogeny, within-litter genetic and epigenetic variances, instead of means or medians, were compared between two developmental stages. The variance among siblings within a litter would remain constant throughout development if the genome and epigenome are permanently established after fertilization. Although tissues at different developmental stages from same individuals cannot be obtained here, litter-based analyses among populations provide an alternative method. Strikingly, we observed lower genetic and epigenetic variances in nearly all 98 litters of day-8 (E8) embryos compared with tissues in 93 litters of 6-week adult offspring (Figure 2 and S1). The differences of most within-litter variances between these two developmental stages were highly significant in all experimental groups (p<0.0004, Data S1). Changes of the variances support that *rDNA* structure and methylation are altered during ontogenic progression from the E8 embryo stage to 6-week adult mice. Furthermore, intraperitoneal injection of Cr(III) or acid saline in the fathers often modified the within-litter variances of the *rDNA* genetic and/or epigenetic traits (Data S1). One or more *rDNA* haplotypes were targeted for modulation of genetic diversity among siblings at various stages of development following paternal treatment with Cr(III) or acid saline and the E8 embryo stage was most frequently affected. In contrast, modifications of *rDNA* methylation diversity among siblings, including increases in E8 embryos in both genders and in female adult livers as well as a decrease in male adult lungs, were primarily observed in offspring from acid saline-treated fathers. These findings demonstrated that the genome is a dynamic molecule, constantly modified at genetic and epigenetic levels in response to normal ontogeny and further influenced by paternal exposures.

### Haplotype-dependent methylation

**Figure 3:**
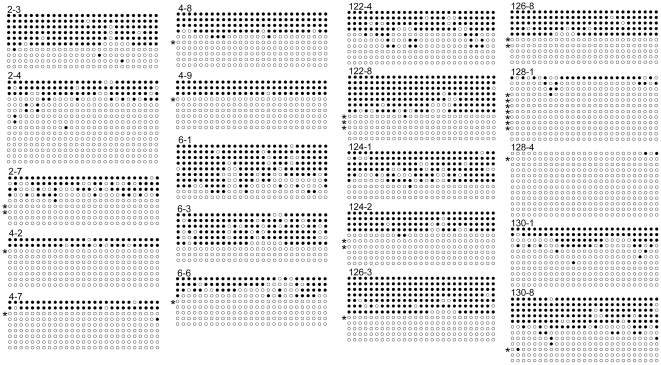
Bisulfite-sequencing of the 27 CpG sites in the *rDNA* spacer promoter region. A total of 20 E8 embryos, identified by hyphened numbers, were examined (filled circle: methylation). *Denotes the T haplotype identified by the −2214 G/T SNP in Figure 1A.

Since only a fraction of *rDNA* repeats is methylated [16], we examined possible haplotype-specific methylation patterns. There were significant associations of T haplotype with hypomethylation as evidenced by population average regression slopes for all tissues (Data S2), which were subsequently confirmed by bisulfite-sequencing (Figure 3). The T-associated hypomethylation in adult sperm also provides a validation to our previous finding in another set of sperm [17]. Similar haplotype-dependent methylation was further detected for CGC (negative association) and CCC (positive association) during normal ontogeny in untreated group except at E8 embryo stage (p<0.003, Data S2). A transit inverse association of CCA with methylation was seen in sperm of untreated mice. Paternal treatment with acid saline, in particular, induced de novo association of methylation with ACC (negative) at both E8 embryo and 6-week adult stages, and with CCA (positive) in adult tissues. The effects of Cr(III) on ACC and CCA haplotypes were strictly limited to female lungs. Taken together, these findings suggest that selected haplotype sequences or SNPs determine the degrees of *rDNA* methylation. Such haplotype-related methylations are modifiable during development and following paternal treatment with Cr(III) or acid saline. It is likely that haplotype-dependent methylation is regulated at multiple levels, including promoter sequence and other yet-to-be identified mechanisms.

### Unequal *rDNA* genotype and epigenotype among tissue lineages

**Figure 4:**
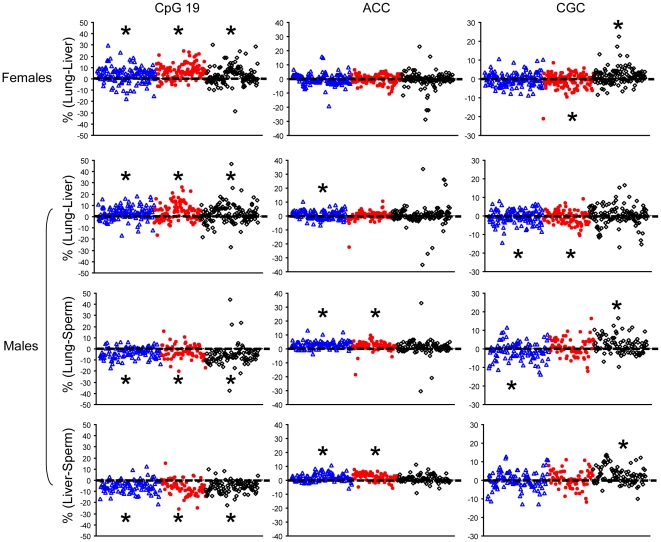
Genetic and epigenetic differences among adult offspring tissues. The arithmetic differences (y-axis) of *rDNA* sequence variant frequency (%, ACC and CGC) and CpG19 methylation (%) between two 6-week adult tissues of the same animals were plotted. Deviations of the differences from a reference line, of an identity with zero difference, were frequently detected in 3 paternal treatment lineages (blue: acidic saline; red: Cr; black: untreated). *Denotes statistical significance in paired difference between tissues of the same mice in each treatment group and identifies positive/negative mean differences by its location above/below the zero reference line (see Data S3 for p values). The standard error lines are omitted to provide clear view of the scatter plot.

The above evidence of *rDNA* rearrangement and methylation change during developmental progression from E8 embryos to 6-week adult mice prompted us to determine whether such genetic and epigenetic modifications also occur in differentiation of tissue lineages. Indeed, direct comparisons of tissues from individual mice revealed that frequencies of several haplotypes and methylation levels of the *rDNA* repeats are often unequal among fully differentiated tissues (p<0.005, Data S3), recapitulating the dynamic nature of the *rDNA* repeats. The shifts of the genetic and epigenetic differences between tissue pairs, away from the zero reference lines, were clearly demonstrated (Figure 4 and S2). Many mice (acidic saline: 30.3–58.8%; Cr: 23.6–62.1%; untreated: 43.7–70.5%) showed tissue-to-tissue difference of more than 5% unit in frequency of at least one haplotype or in *rDNA* methylation, a chosen threshold larger than the standard deviation of 1.25% to 3.97% from duplicate assays, as described above. The tissue-differential *rDNA* methylation mirrors a report showing variably methylated regions among tissues in mice and humans [18]. Frequent observation of differential genotype between sperm and two somatic cells (lung and liver) is consistent with genetic rearrangement contributed by meiotic recombination during spermatogenesis. These ontogeny-related genetic and epigenetic diversities were further modified by paternal injection of Cr(III) or acidic saline (Data S3). One or more haplotypes were targeted for enlargement of the differences among tissues except lung versus liver in male mice. The paternal effects were also observed for *rDNA* methylation in female lung/liver and male lung/sperm pairs.

**Figure 5:**
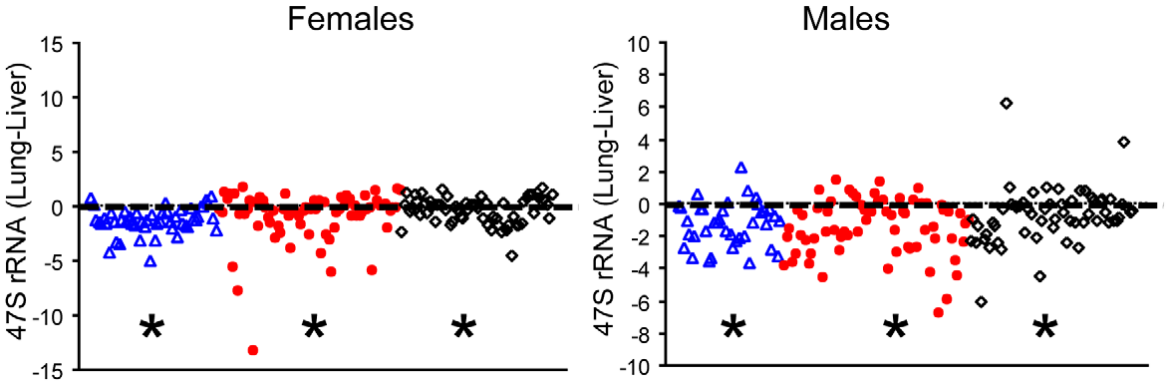
Differential *rRNA* level between tissues. The scatter plot of arithmetic difference (y-axis) between lungs and livers of individual mice in 3 paternal treatment lineages shows higher *rRNA* level in livers compared with lungs (blue: acidic saline; red: Cr; black: untreated). *Denotes p<0.003 (paired T test) and identifies positive/negative mean differences by its location above/below the zero reference line.

It is noteworthy that sperm had the highest *rDNA* methylation level among three tissues, followed by lung and liver (Figure 4). The high *rDNA* methylation in sperm is consistent with decreases of hypomethylated haplotypes (T, ACC, and/or CGC, Data S2) but increases of those associated with hypermethylation (CCA and/or CCC). In contrast, the low *rDNA* methylation in livers was accompanied with more hypomethylated but less hypermethylated haplotypes compared with lungs and sperm. Low *rDNA* methylation is known to enact *rRNA* transcription [19]. As predicted, livers expressed higher levels of the *47S* pre-*rRNA* than lungs from same mice (Figure 5).

### Differential transcription and methylation of *rDNA* haplotypes

**Figure 6:**
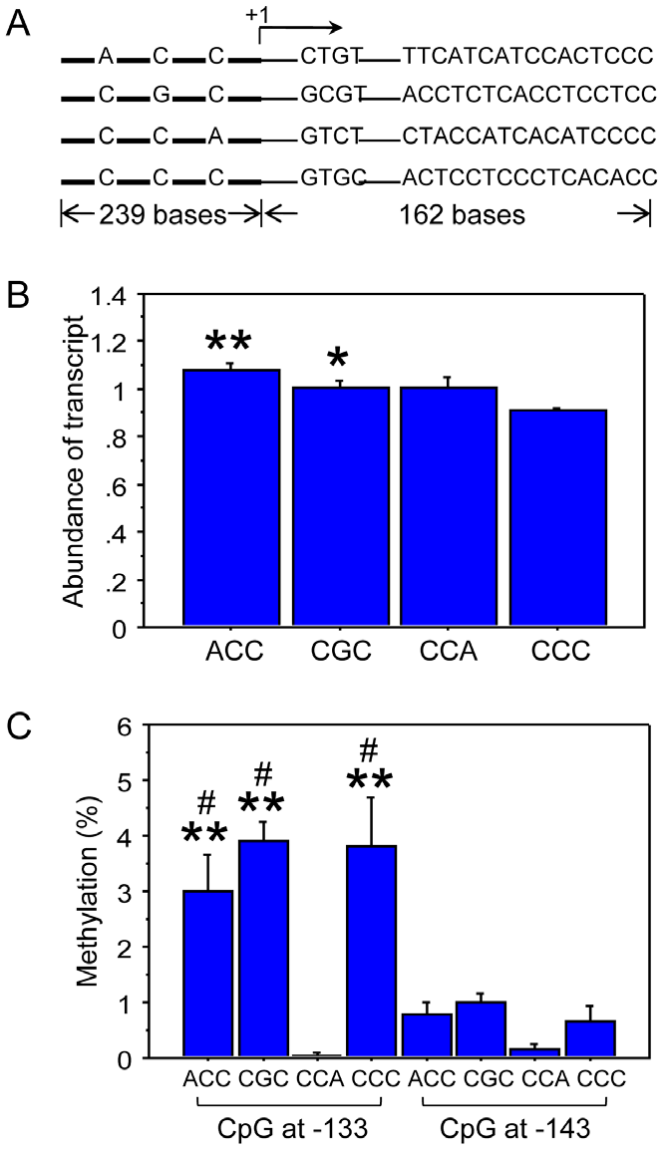
Haplotype-related *rRNA* transcription and *rDNA* methylation *in vitro*. (A) Four haplotype constructs carrying unique SNPs in the *rDNA* promoter region (bold line) and tetranucleotide identifications in *rRNA* transcripts (+1: transcription start site). The differential 3′-end sequences provide priming sites for haplotype-specific enrichment. (B) The relative abundance of each haplotype transcript corrected by that of *rDNA* template (4 independent measurements, *p<0.05, **p<0.01 compared with the CCC, unpaired T test). (C) The level of methylation in each of 4 haplotypes from the same sample in *in-vitro* transcription assay after subtraction of background that was measured in originally unmethylated haplotype construct (4 independent measurements, **p<0.01 compared with the CCA, ^#^p<0.05 compared with corresponding haplotype at the −143 site).

Preferential selection of hypomethylated *rDNA* haplotype and more *rRNA* level in livers, compared with lungs, suggests that the SNPs in the *rDNA* gene promoter may in theory control *rRNA* transcription. *In vitro* transcription was initiated by incubating DNA constructs with identifiable SNPs for 4 haplotypes (ACC, CGC, CCA, and CCC) in a mouse nuclear extract to test the hypothesis (Figure 6A). The relative level of *rRNA* transcript for each haplotype was normalized with that of *rDNA* template, remained at low quantity and amplified from the same samples, to correct for variation of pyrosequencing signal among the 4 identification sites (Figure 6B). The mean corrected ratios, 1.08, 1.00, 1.00, and 0.91 (equivalent to 27.0%, 25.0%, 25.0%, and 22.8%) were obtained for ACC, CGC, CCA, and CCC, respectively, indicative of the relative abundance of *rRNA* transcripts in the following order, ACC>CGC = CCA>CCC. The difference, either ACC or CGC versus CCC, reached statistical significance. This is consistent with the above observation that livers tend to maximize *rRNA* output by selecting more CGC haplotype but less CCC than lungs (Figure 4 and S2).

It has been shown that methylation of naked *rDNA* without chromatin assembly did not alter *rRNA* transcription [19]; however, the haplotype constructs provide useful materials to test if methylation is directly regulated by DNA sequence. Two CpG sites in the *rDNA* promoter of each haplotype after *in vitro* transcription reaction were examined and intriguingly the level of methylation was dependent on the CpG site and haplotype sequence (Figure 6C). Preferential methylation was detected at −133 CpG compared with the −143 site and the CCA haplotype was nearly unmethylated. Methylation of the single CpG site at −133 is known to silence *rRNA* transcription *in vivo* or *in vitro* with chromatin assembly [19]. A slight, about 3–4%, but significant methylation of ACC, CGC, and CCC at the −133 CpG in nuclear extract suggests that these haplotypes are directly accessible to methyltransferase(s) and subjected to methylation modification. In contrast, the CCA may follow different mechanism. This prediction is supported by the above observation of nonlinear association of methylation with CCA, namely, negative association in sperm of untreated mice, no association in E8 embryos and adult tissues of untreated group, but positive association in adult offspring after paternal treatment with acid saline (Data S2).

**Figure 7:**
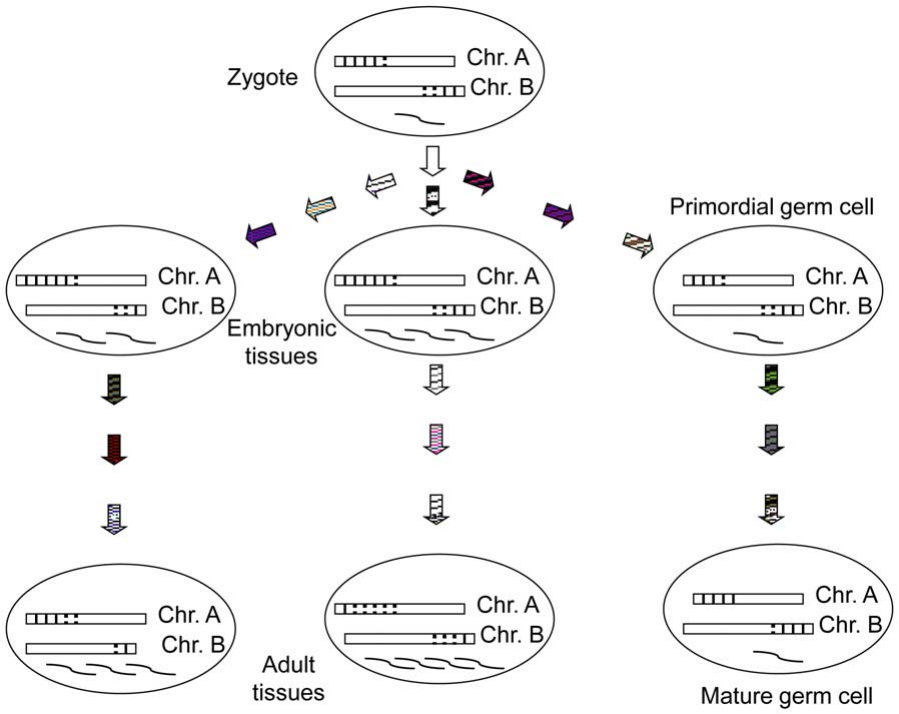
Proposed model for ontogeny-induced *rDNA* rearrangement, methylation, and transcription. Different cell lineages undergo serial stages of *rDNA* genetic/epigenetic and transcriptional modifications, as indicated by vertical bars for two haplotypes (broken: hypomethylated; solid: hypermethylated) in multiple chromosomes (Chr.) and helical lines, respectively. These *rDNA* activities, including copy number changes, are driven by distinct stage-dependent ontogenic stimulants, represented by arrows with diverse textures.

We demonstrated here that the genome is dynamic throughout normal ontogenic processes and is also subjected to modulation by paternal exposures. The consequences of *rDNA* rearrangement during ontogenic cell division and differentiation potentially include changes in *rDNA* methylation and *rRNA* transcription. Observations of tissue-specific *rDNA* haplotype frequencies, *rDNA* methylation, and *rRNA* transcription provide further evidence that the *rDNA* rearrangement may participate in normal ontogeny, as proposed in Figure 7. The role of *rRNA* biogenesis in cell lineage decision has been documented in various model systems. It has been shown that transcription of the *rDNA* to *rRNA* is a rate-limiting factor for ribosomal biogenesis [20], which is also required for cell cycle progression in several cell lineages [21]. Correct *rRNA* processing is essential for regulation of renewals and differentiations of asymmetrically segregated germline stem cells and neuroblasts in *Drosophila* [22]. The *rDNA* gene has been also associated with cancers [23], [24], aging [25], neurodegenerative diseases [26], [27], and psychiatric disorders [28]. Unequal sister chromatid recombination, initiated by RNA polymerase II transcription within the intergenic region of the *rDNA*, has been shown to trigger amplification of *rDNA* copies in yeast [29]. Similar hypomethylation-induced RNA polymerase II-mediated increase of epichromosomal *rDNA*, indicative of amplification, was also seen in human cells [24]. Our observations of serial epigenetic and transcriptional changes after *rDNA* rearrangement support the key function of the *rDNA* repeats in organismal ontogeny and disease processes.

## Materials and Methods

### Ethics statement

All animals used in this research project were cared for and used humanely according to the following policies: The *U.S. Public Health Service Policy on Humane Care and Use of Animals* (1996) and the U.S. Government *Principles for Utilization and Care of Vertebrate Animals Used in Testing, Research, and Training* (1985). The animal studies (01-32H, 04-037H, 04-037J, 04-037K, and 04-037M) were approved by National Cancer Institute-Frederick Animal Care and Use Committee.

### Animal treatment

Outbred male Cr:NIH Swiss mice were obtained from the National Cancer Institute-Frederick Animal Production Area at 6 weeks of age, housed for 2 weeks, and were injected intraperitoneally with 1 mmol/kg CrCl_3_·6H_2_O in 0.5 ml 0.9% NaCl adjusted to pH 4.0 with NaOH or HCl-acidified saline alone (pH 4). The glucose-lowering efficiency of the current Cr(III) regimen was validated (Figure S3). For acquisition of sperm from direct exposure, two separate series of 20 and 25 males respectively were treated, and 2 wks later the epididymides from mice (41 in Cr(III), 44 in acidic saline, and 43 in untreated groups) were frozen for sperm isolation. For the preconceptional studies, the male mice were treated with Cr(III), acidic saline, or nothing, as described above, and mated with untreated females 2 weeks after treatments. In order to minimize batch-related effects, such as seasonal changes, suggested by previous investigators [30], several experimental batches were utilized. A total of 876 E8 embryos were collected from 26 litters in Cr(III) group, 36 litters in acidic saline group, and 36 litters in no treatment group, as two experimental batches over one year. Tissues (lung, liver, and epididymides) from 6-week adult offspring were obtained from three different experimental series except epididymides from only two, carried out over 18 months. A total of 758 offspring from 31 Cr(III) group litters, 32 acidic saline group litters, and 30 no treatment group litters comprised this part of the study.

### DNA extraction and bisulfite treatment

DNAs of entire E8 embryos and 6-week old offspring lung and liver were extracted using Easy-DNA kits (Invitrogen, Carlsbad, CA). Epididymides were minced in phosphate-buffered normal saline. After precipitation of tissue debris by gravity, the sperm in the supernatant were transferred for subsequent sonication to destroy cells of non-sperm origin. Sperm heads were then pelleted by centrifugation at 1,000× g and DNA was extracted using proteinase K with the presence of dithiothreitol, as described previously [11], [17]. About 500 ng of genomic DNA were used for bisulfite treatment according to a previously established protocol [11], [17].

### Quantification of DNA methylation and sequence variants

The bisulfite-modified sense strands of the *rDNA* spacer promoter (GenBank Accession BK000964) were amplified by real-time polymerase chain reactions (PCRs), using forward primer: ggaagtgtttgtggtgagg and a biotinylated reverse primer: biotin-caccaaccctaacatttttcc. In brief, 5 or 10 µl of modified genomic DNA was used in a final 50 µl reaction containing 0.5 µM primers and 1× QuantiTect SYBR Green PCR mix (Qiagen, Valencia, CA). PCR was performed with an initial denaturation at 95°C for 15 minutes followed by 35 cycles of denaturation (94°C, for 10 s), annealing (50°C, 30 s), and extension (72°C, 30 s) in a Chromo4 real-time system (Bio-Rad, Hercules, CA). Regions of the *rDNA* spacer and main gene promoters were amplified using forward/reverse primers, biotin-gcgcgtgagcgatctgta/ctggtcgcctcaccacag and tctgaggccgagggaaagcta/biotin-ggaaagtgacaggccacagagaat, respectively. The PCR conditions were the same as described above, except that 50 ng unmodified DNA, annealing at 60°C, and amplification for a total of 30 cycles were applied. Entire PCR products were used directly for pyrosequencing to obtain representative estimation from >200 *rDNA* copies in mouse genome. The percentages of methylation in the spacer promoter and of SNPs in both spacer- and main gene promoters were quantified by pyrosequencing using 4 primers in the PyroMark MD system (Biotage, Foxboro, MA). For DNA methylation, initial assay covered 9 CpG sites as described previously [17]. Since the levels of methylation were parallel across these sites among the three treatment groups, subsequent assays focused on the 19–23 CpG sites. These 5 CpG sites were examined by the gattagggtgataggag primer in a single reaction. The T/G SNP at −2214 in the spacer promoter, reported before [17], was measured by the ctcgatcaaccatacaaa primer. The same PCR products from the main gene promoter region were used for two pyrosequencing reactions. The ggaaagctatgggcg primer was used to interrogate SNPs A/C at −218 and C/G at −178, and the tttgggccacctcc primer for C/A SNP at −104. These sequence variants were deposited in GenBank (see also Figure 1B). The frequencies of ACC, CGC, and CCA were obtained directly from the percentages of A/C, C/G, and C/A SNPs, respectively. The CCC frequency was calculated by subtraction of the total of ACC, CGC, and CCA percentages from 100. Independently repeated PCR and pyrosequencing were performed to acquire duplicated values for statistical analyses.

### Cloning and dideoxy DNA sequencing

Cloning was performed using a TOPO Cloning kit (Invitrogen Co.) to obtain individual sequence clones for Sanger's dideoxy DNA sequencing. The bacteria colonies carrying plasmids of the PCR product inserts flanked by the primers, gagctttggatcttattttttttttaattttttct/gcgacagacccaagccagtaaa for unmodified DNAs or gtttgaggttgagggaaagtta/tcaatacctatctccaaatccaata for bisulfite-modified templates, were positively selected in culture plates with the presence of ampicillin and kanamycin antibiotics.

### Gender typing

Genders of the E8 embryos were determined by real-time PCR using primers gtgagaggcacaagttggc/ctctgtgtaggatcttcaatctct for the *SRY* gene (GenBank NM_011564) on the Y chromosome and primers aactaaatcaaagactcaagcatg/tgtgcagagtgataaatatggatc for the *X-inactivation center region* (GenBank AJ421478) on the X chromosome. The presence of X and/or Y chromosome(s) was detected by melting curves in the Chromo4 system (Bio-Rad).

### Quantification of the *47S* pre-*rRNA*

Total RNA was isolated with the RNeasy Mini kit (Qiagen) according to the manufacturers' protocols. DNase treatment was performed to remove residual DNA from the RNA samples by use of the recombinant DNase I as specified in the protocol supplied with the Ambion DNA-free Kit (Applied Biosystems, Foster City, CA). The treated samples were purified and a 40 ng total RNA was used for reverse transcription (RT) and quantitative PCR, as described previously [31]. In brief, RT was carried out using SuperScript III (Invitrogen) and gene-specific primers according to the manufacturer's protocol. The primer used for RT determination of a 5′ *rRNA* Leader sequence region, indicative of the *47S* pre-*rRNA* level, was gagacaaacctggaacg. PCR primer pair was: gtggagagtcccgagtactt/ggggcaagacagttactgata. One universal reference total RNA was included in every PCR run to obtained relative level of the *47S* pre-rRNA before comparison across all samples.

### *In vitro* transcription

Four haplotype constructs were generated by PCR from pMA plasmids (GENEART, Regensburg, Germany) containing modified *rDNA* sequences (−248 to +87, see Figure 1A and 6A) flanked by AscI and PacI restriction sites. Modifications of internal sequences (C to G at +6, A to C at +33, and deletion of AAA at +44 to +46 for all; substitutions of CCCT at +51 to +54 with TCTGTG, TGCGTG, TGTCTG, and TGTGCG for ACC, CGC, CCA, and CCC haplotype, respectively) were introduced to maximize specificity for pyrosequencing assay. The final products of 401 bps were amplified using a universal upper primer (gccgagggaaagctatgg) and a haplotype-specific lower primer (gggagtggatgatgaaactggaaagcgggcagtg for ACC; ggaggaggtgagaggtactggaaagcgggcagtg for CGC; ggggatgtgatggtagactggaaagcgggcagtg for CCA; ggtgtgagggaggagtactggaaagcgggcagtg for CCC). Equal amounts of the 401-bp haplotype constructs (10 ng each) were incubated with 25 ug P19 mouse teratocarcinoma nuclear extract (Active Motif, Carlsbad, CA) supplemented with 0.5 mM ribonucleotides, 5 mM MgCl_2_, 0.1 mM EDTA in a 20 ul reaction for 60 minutes at 30°C, as described previously [32]. Total RNAs were purified by addition of TRIzol Reagent (Invitrogen), followed by spin-column capture using MEGAclear kit (Ambion, Austin, TX) and subsequently treated with DNase I (DNA-free kit, Ambion). The *rRNA* transcripts were reverse-transcribed utilizing SuperScript III (Invitrogen) and the actggaaagcgggcagtg primer. PCR products, amplified with gacgctgtcctttccctatt/biotin-gccagtcttgtgctccatta, were pyrosequenced using the cactacaggacactatgaga primer to quantify C/G, C/T, C/G, and C/T haplotype-specific SNPs at +49 to +52. Residual *rDNA* constructs from each sample were also amplified after omitting reverse transcriptase in RT reaction but with intensity of >30-fold lower than the *rRNA* transcripts. For CpG methylation, a haplotype-specific primer (gggagtggatgatgaaattg for ACC, ggaggaggtgagaggtattg for CGC, ggggatgtgatggtagattg for CCA, or ggtgtgagggaggagtattg for CCC) and a common lower primer (accaattattcctttaaaatcc), were used to enrich individual haplotype from bisulfite-modified DNA with 7 cycles of PCR in separate reactions. A 1/100 dilution of PCR products was further amplified for 34 cycles using an upper primer (tggaagttatatttggggaggt for ACC, CGC, and CCC haplotype; tggaagttatatttgtggaggt for CCA) and the biotin-accaattattcctttaaaatcc lower primer. Pyrosequencing primer, tggtttaaaaatgattttat, was used to interrogate methylation at −133 and −143 CpG sites.

### Statistical analysis

Linear mixed-effects models were used to test for treatment differences [e.g., untreated, acidic saline, and Cr(III)] and to account for the effects of covariates, including sex of offspring, litter size, and experimental batch effects on response endpoints [33]–[35]. These models included both random and fixed effects. Random effects for litter were included in the model to account for correlation of responses between mice belonging to the same litter. Random effects for mouse within litter were also included to account for correlation of the replicate measurements made on the same mouse. The fixed effects included an analysis-of-variance (anova) component to model the effects of the treatments. F statistics were used to test for homogeneity of the mean responses across the treatments, and t statistics were used to make additional pairwise comparisons between the treatment means. Further analysis methods included correlation and regression, analysis of variance (anova), t-tests, Wilcoxon tests and tests for variance homogeneity. Probability values less than 0.05 were considered to be significant. All probability values were two sided.

## Supporting Information

Figure S1 **Increase of littermate-to-littermate variation during ontogeny.** Within-litter variation, represented by error bar, of *rDNA* sequence variant frequencies (CGC, CCA, and CCC) widened from E8 embryos to 6-week adult tissues in 3 paternal treatment lineages (genders combined; blue: acidic saline; red: Cr; black: untreated). The plots are displayed as mean±1x standard deviation to illustrate the degree of variation.
(TIF)

Figure S2 **Genetic differences among adult offspring tissues.** The arithmetic differences (y-axis) of *rDNA* sequence variant frequencies (%, T, CCA, and CCC) between two 6-week adult tissues of the same animals were plotted. Deviations of the differences from a reference line, of an identity with zero difference, were frequently detected in 3 paternal treatment lineages (blue: acidic saline; red: Cr; black: untreated). *Denotes statistical significance in paired difference between tissues of the same mice in each treatment group and identifies positive/negative mean differences by its location above/below the zero reference line (see Data S3 for p values). The standard error lines are omitted to provide clear view of the scatter plot.
(TIF)

Figure S3 **Cr(III)- and acidic saline-induced acute phenotypic changes in male mice.** Body weight curves of breeding males (10 mice each) and serum glucose (19 or 20 mice each) of males 2 weeks after treatment (blue: acidic saline; red: Cr; black: untreated). *Denotes p<0.01, two-sample T test, in reference to untreated group. Intraperitoneal injection of Cr(III) at week 8 resulted in acute weight loss. Acidic saline did not induce any weight loss or change of serum glucose. Body weights also dropped at the one week breeding period commencing at week 10 for all three experimental groups but recovered at week 12. The same results were reproduced from other batches of male mice.
(TIF)

Data S1 **Equality of within-litter variances among E8 embryos and 6-week adult offspring tissues, and pairwise comparisons of treatments.**
(DOC)

Data S2 **Mixed-effects model of regression slopes between CpG methylation and sequence variants.**
(DOC)

Data S3 **Paired T test of tissue versus tissue and mixed-effects model of pairwise comparisons of treatments.**
(DOC)
