# Supplementary figures and images for: Ontogeny-Driven rDNA Rearrangement, Methylation, and Transcription, and Paternal Influence
Source: PLoS One. 2011 Jul 12;6(7):e22266. doi: 10.1371/journal.pone.0022266 (PMC3134480; doi:10.1371/journal.pone.0022266)

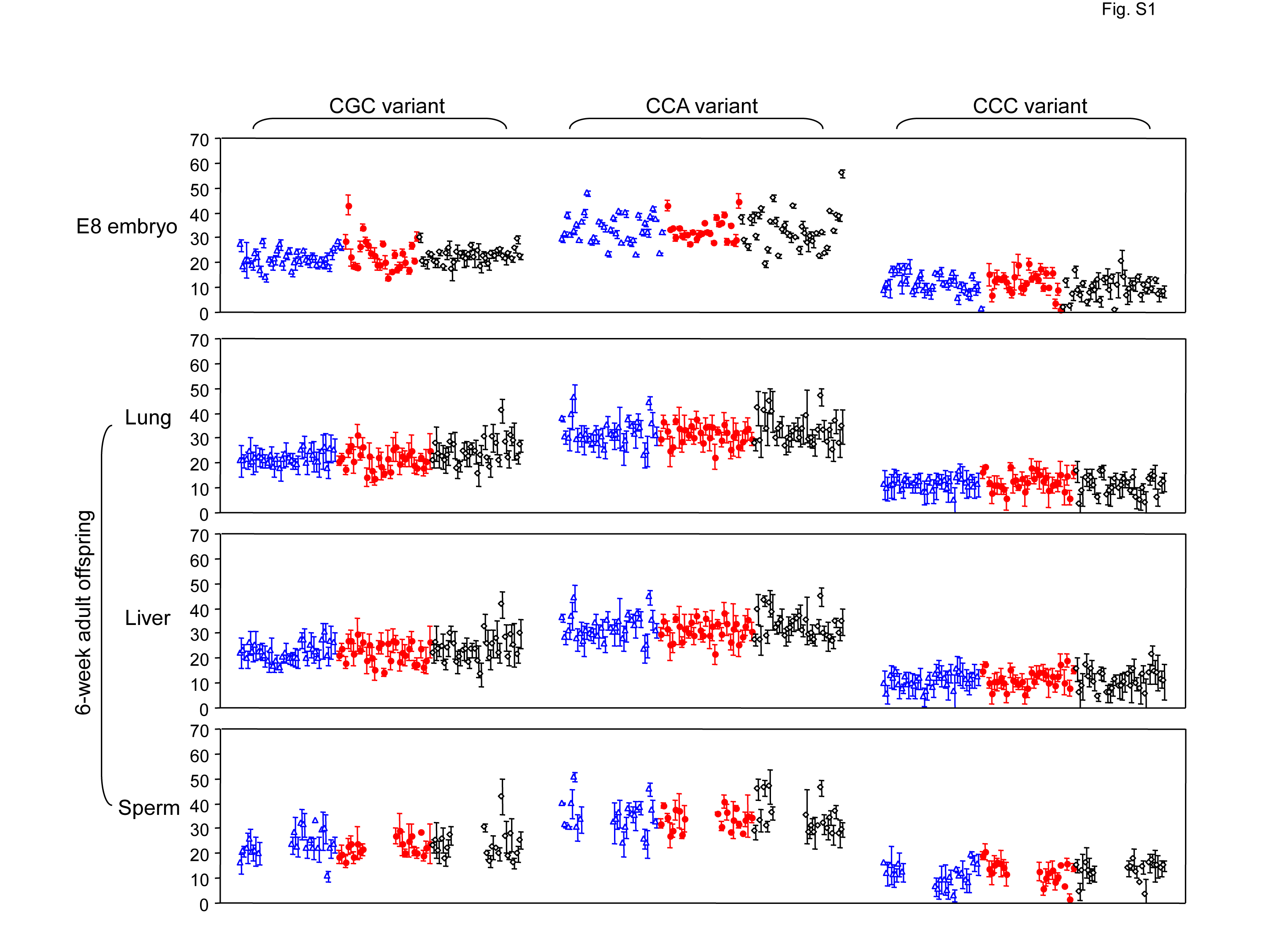

Supplement: Figure S1 — Increase of littermate-to-littermate variation during ontogeny. Within-litter variation, represented by error bar, of rDNA sequence variant frequencies (CGC, CCA, and CCC) widened from E8 embryos to 6-week adult tissues in 3 paternal treatment lineages (genders combined; blue: acidic saline; red: Cr; black: untreated). The plots are displayed as mean±1x standard deviation to illustrate the degree of variation. (TIF) [file pone.0022266.s001.tif]

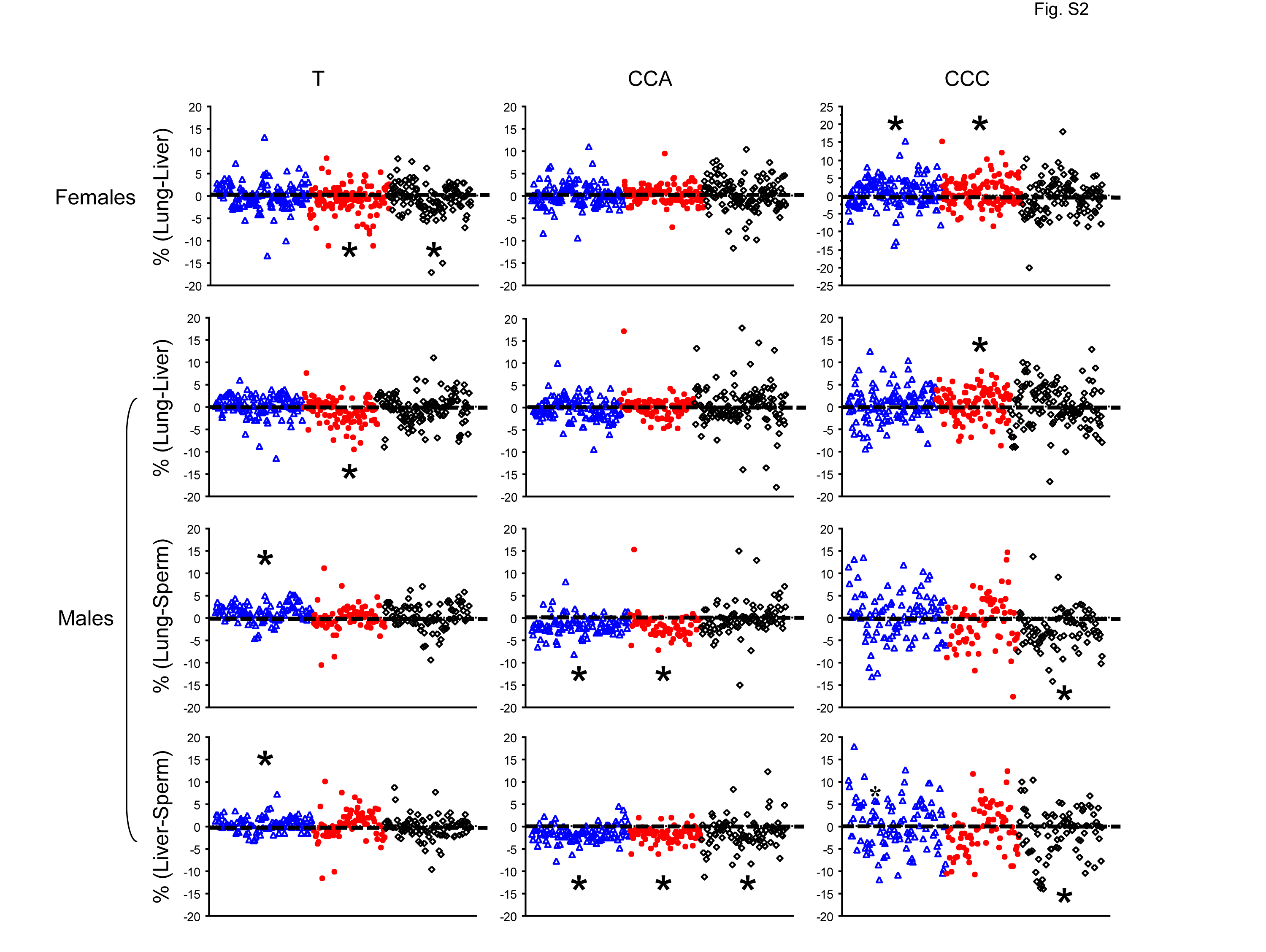

Supplement: Figure S2 — Genetic differences among adult offspring tissues. The arithmetic differences (y-axis) of rDNA sequence variant frequencies (%, T, CCA, and CCC) between two 6-week adult tissues of the same animals were plotted. Deviations of the differences from a reference line, of an identity with zero difference, were frequently detected in 3 paternal treatment lineages (blue: acidic saline; red: Cr; black: untreated). *Denotes statistical significance in paired difference between tissues of the same mice in each treatment group and identifies positive/negative mean differences by its location above/below the zero reference line (see Data S3 for p values). The standard error lines are omitted to provide clear view of the scatter plot. (TIF) [file pone.0022266.s002.tif]

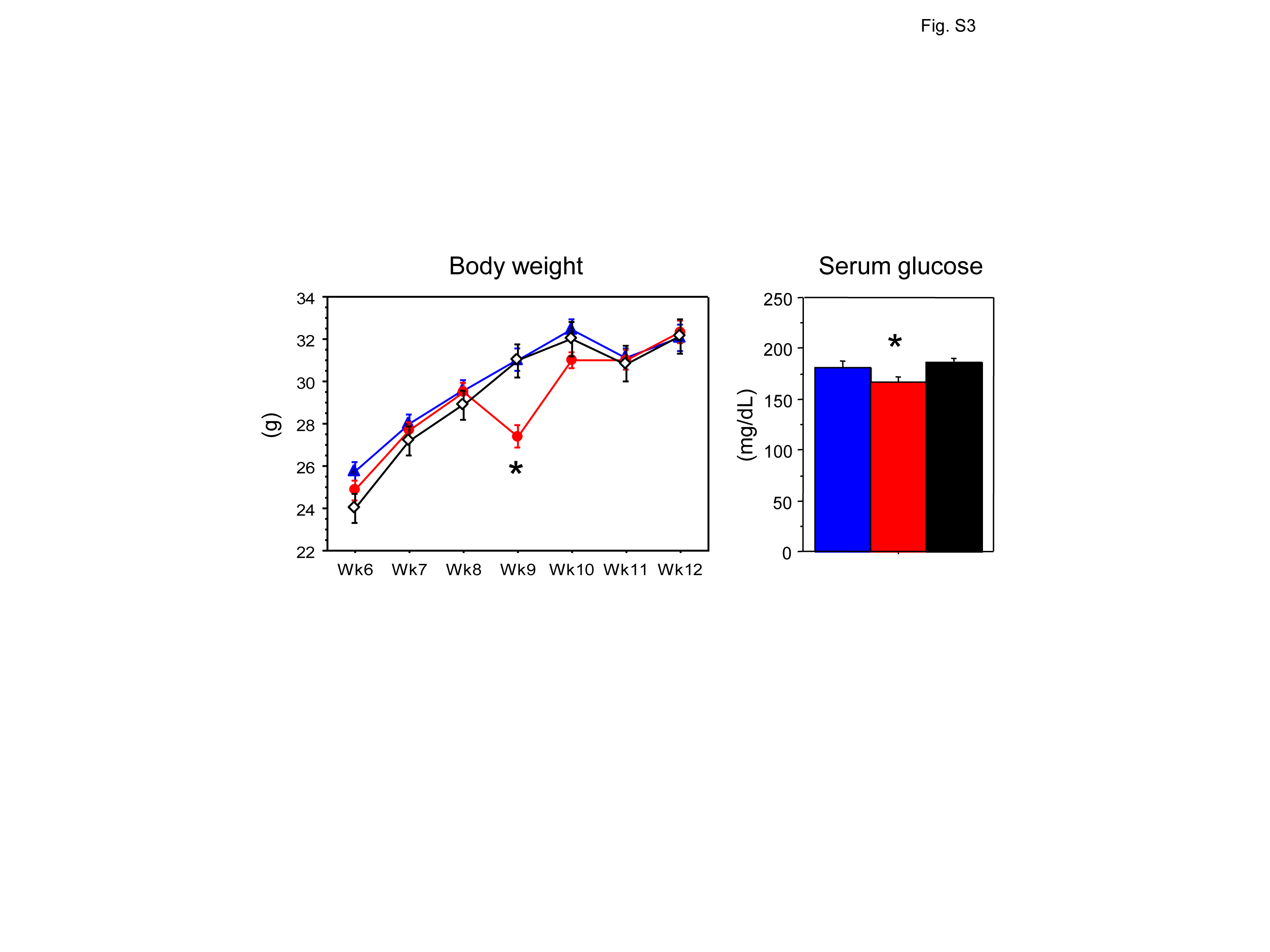

Supplement: Figure S3 — Cr(III)- and acidic saline-induced acute phenotypic changes in male mice. Body weight curves of breeding males (10 mice each) and serum glucose (19 or 20 mice each) of males 2 weeks after treatment (blue: acidic saline; red: Cr; black: untreated). *Denotes p<0.01, two-sample T test, in reference to untreated group. Intraperitoneal injection of Cr(III) at week 8 resulted in acute weight loss. Acidic saline did not induce any weight loss or change of serum glucose. Body weights also dropped at the one week breeding period commencing at week 10 for all three experimental groups but recovered at week 12. The same results were reproduced from other batches of male mice. (TIF) [file pone.0022266.s003.tif]
