# Supplementary material for: Ontogeny-Driven rDNA Rearrangement, Methylation, and Transcription, and Paternal Influence
Source: PLoS One. 2011 Jul 12;6(7):e22266. doi: 10.1371/journal.pone.0022266 (PMC3134480; doi:10.1371/journal.pone.0022266)
Supplement: Data S1 — Equality of within-litter variances among E8 embryos and 6-week adult offspring tissues, and pairwise comparisons of treatments. (DOC) [file pone.0022266.s004.doc]

Data S1. Equality of within-litter variances among E8 embryos and 6-week adult offspring tissues, and pairwise comparisons of treatments. (red: p<0.05, with variance ratio less than 1; yellow: p<0.05, with variance ratio greater than 1).

I. F test for the equality of within-litter variance between day-8 embryo and 6-week adult offspring

i. E8 embryo versus 6-week adult offspring lung

**Group Untreated Acid Saline Cr(III)**

Embryo lung p-value Embryo lung p-value Embryo lung p-value

### All mice

**CPG19** 9.3207 44.2216 <.0001 12.8393 37.4578 <.0001 8.2192 41.0893 <.0001

**CPG20** 8.2449 40.0797 <.0001 10.8893 33.1145 <.0001 6.4366 37.1580 <.0001

**CPG21** 7.7177 37.6314 <.0001 11.1641 32.3393 <.0001 6.9055 36.8285 <.0001

**CPG22** 11.0137 47.0686 <.0001 14.9329 37.1367 <.0001 8.6987 43.6791 <.0001

**CPG23** 12.8684 53.8829 <.0001 20.9764 46.1605 <.0001 14.1228 52.5630 <.0001

**T**  0.6784 11.5945 <.0001 1.2255 13.9457 <.0001 1.8703 9.3488 <.0001

**ACC**  1.2945 34.6918 <.0001 1.0267 23.9244 <.0001 1.6879 28.9068 <.0001

**CGC** 3.3711 14.2384 <.0001 3.6937 14.7962 <.0001 3.5074 12.5813 <.0001

**CCA** 1.7937 19.8800 <.0001 0.8140 16.3059 <.0001 1.2465 14.0782 <.0001

**CCC** 5.6998 9.9042 <.0001 3.4607 12.9572 <.0001 4.1880 11.7392 <.0001

### Females

**CpG19** 9.5455 36.6615 <.0001 12.8072 39.9868 <.0001 8.7569 45.1890 <.0001

**CpG20** 8.2276 30.2968 <.0001 11.3473 34.3497 <.0001 6.8830 38.7610 <.0001

**CpG21** 7.8074 28.6579 <.0001 11.6349 34.7673 <.0001 6.7569 37.6404 <.0001

**CpG22** 9.6014 36.6161 <.0001 16.8016 40.5210 <.0001 9.4329 47.2522 <.0001

**CpG23** 11.4657 42.3750 <.0001 21.9226 51.7810 <.0001 14.8820 56.4062 <.0001

**T**  0.7520 12.0727 <.0001 1.4831 14.2519 <.0001 1.5398 8.5560 <.0001

**ACC**  1.7770 33.6996 <.0001 1.1218 26.2819 <.0001 2.2081 27.6693 <.0001

**CGC**  4.5030 13.2447 <.0001 4.7084 13.7603 <.0001 2.5255 14.5607 <.0001

**CCA**  1.9174 19.0109 <.0001 0.9329 16.9206 <.0001 1.4980 13.4160 <.0001

**CCC** 7.6891 11.3477 0.0214 4.0414 12.5055 <.0001 3.4020 11.7322 <.0001

### Males

**CpG19** 9.1654 51.8857 <.0001 12.9577 34.8895 <.0001 7.6663 36.5203 <.0001

**CpG20** 8.3098 49.8363 <.0001 10.4425 31.9309 <.0001 5.9736 35.4368 <.0001

**CpG21** 7.6791 46.5867 <.0001 10.7044 29.8033 <.0001 7.1539 35.9967 <.0001

**CpG22** 12.3985 57.5566 <.0001 12.9074 33.6099 <.0001 7.9174 39.7804 <.0001

**CpG23** 14.2543 65.4448 <.0001 20.0371 40.1653 <.0001 13.3677 48.3933 <.0001

**T**  0.6137 11.2245 <.0001 0.9408 13.7118 <.0001 2.2823 10.3678 <.0001

**ACC**  0.8513 35.9033 <.0001 0.9253 21.4510 <.0001 1.0878 30.6123 <.0001

**CGC** 2.3400 15.2875 <.0001 2.5647 16.0783 <.0001 4.7122 10.3751 <.0001

**CCA** 1.6888 20.8577 <.0001 0.6841 15.7417 <.0001 0.9608 14.9717 <.0001

**CCC** 3.8984 8.5901 <.0001 2.8228 13.5552 <.0001 5.1668 11.8489 <.0001

ii. E8 embryo versus 6-week adult offspring liver

**Group Untreated Acid Saline Cr(III)**

Embryo liver p-value Embryo liver p-value Embryo liver p-value

#### All mice

**CPG19** 9.3207 38.3236 <.0001 12.8393 47.6963 <.0001 8.2192 43.2657 <.0001

**CPG20** 8.2449 30.2713 <.0001 10.8893 40.2842 <.0001 6.4366 36.1853 <.0001

**CPG21** 7.7177 29.7492 <.0001 11.1641 38.3878 <.0001 6.9055 33.3939 <.0001

**CPG22** 11.0137 33.6315 <.0001 14.9329 44.7299 <.0001 8.6987 41.7459 <.0001

**CPG23** 12.8684 43.6704 <.0001 20.9764 57.1711 <.0001 14.1228 53.1371 <.0001

**T**  0.6784 11.8078 <.0001 1.22554 16.3496 <.0001 1.8703 12.9788 <.0001

**ACC**  1.2945 25.2420 <.0001 1.02671 23.9556 <.0001 1.6879 26.8623 <.0001

**CGC** 3.3710 17.2848 <.0001 3.69371 14.0377 <.0001 3.5074 13.2342 <.0001

**CCA** 1.7937 13.2305 <.0001 0.81395 15.0564 <.0001 1.2465 13.1852 <.0001

**CCC** 5.6998 13.1624 <.0001 3.46065 13.0166 <.0001 4.1880 8.8037 <.0001

#### Females

**CpG19** 9.5455 33.4457 <.0001 12.8072 50.0186 <.0001 8.7569 42.2592 <.0001

**CpG20** 8.2276 27.2940 <.0001 11.3473 41.3688 <.0001 6.8830 35.1439 <.0001

**CpG21** 7.8074 26.0473 <.0001 11.6349 40.1938 <.0001 6.7569 32.4054 <.0001

**CpG22** 9.6014 29.5763 <.0001 16.8016 47.5251 <.0001 9.4329 40.2060 <.0001

**CpG23** 11.4657 40.4818 <.0001 21.9226 59.8522 <.0001 14.8820 49.2860 <.0001

**T**  0.7520 13.5439 <.0001 1.4831 17.3984 <.0001 1.5398 14.4670 <.0001

**ACC** 1.7770 29.2351 <.0001 1.1218 26.3828 <.0001 2.2081 28.5029 <.0001

**CGC** 4.5030 19.5461 <.0001 4.7084 12.7868 <.0001 2.5255 16.3221 <.0001

**CCA** 1.9174 12.4449 <.0001 0.9329 15.8336 <.0001 1.4980 13.0596 <.0001

**CCC** 7.6891 16.1279 <.0001 4.0414 13.8675 <.0001 3.4020 8.8354 <.0001

#### Males

**CpG19** 9.1654 43.1708 <.0001 12.9577 45.4844 <.0001 7.6663 44.5936 <.0001

**CpG20** 8.3098 33.2449 <.0001 10.4425 39.3566 <.0001 5.9736 37.4665 <.0001

**CpG21** 7.6791 33.3903 <.0001 10.7044 36.6410 <.0001 7.1539 34.6004 <.0001

**CpG22** 12.3985 37.6768 <.0001 12.9074 41.9695 <.0001 7.9174 43.7246 <.0001

**CpG23** 14.2543 46.9716 <.0001 20.0371 54.6322 <.0001 13.3677 57.8975 <.0001

**T**  0.6137 10.2392 <.0001 0.9408 15.3139 <.0001 2.2823 11.3588 <.0001

**ACC**  0.8513 21.6472 <.0001 0.9253 21.4494 <.0001 1.0878 25.1924 <.0001

**CGC** 2.3400 15.2604 <.0001 2.5647 15.5557 <.0001 4.7122 9.7319 0.0004

**CCA** 1.6888 14.0868 <.0001 0.6841 14.3106 <.0001 0.9608 13.4357 <.0001

**CCC** 3.8984 10.4578 <.0001 2.8228 12.1517 <.0001 5.1668 8.8378 <.0001

iii. E8 embryo versus 6-week adult offspring sperm

**Group Untreated Acid Saline Cr(III)**

Embryo sperm p-value Embryo sperm p-value Embryo sperm p-value

**CpG19** 9.1654 34.3649 <.0001 12.9577 39.0266 <.0001 7.6663 28.2143 <.0001

**CpG20** 8.3098 34.2850 <.0001 10.4425 35.3599 <.0001 5.9736 27.1357 <.0001

**CpG21** 7.6791 36.0728 <.0001 10.7044 35.5575 <.0001 7.1539 27.3080 <.0001

**CpG22** 12.3985 35.0602 <.0001 12.9074 39.4420 <.0001 7.9174 27.6717 <.0001

**CpG23** 14.2543 34.9684 <.0001 20.0371 47.4681 <.0001 13.3677 32.6301 <.0001

**T**  0.6137 11.7569 <.0001 0.9408 12.2452 <.0001 2.2823 6.3602 <.0001

**ACC** 0.8513 22.6310 <.0001 0.9253 21.5577 <.0001 1.0878 19.9814 <.0001

**CGC** 2.3400 16.6166 <.0001 2.5647 19.7116 <.0001 4.7122 11.1066 <.0001

**CCA** 1.6888 15.5266 <.0001 0.6841 16.4593 <.0001 0.9608 10.2214 <.0001

**CCC** 3.8984 13.7013 <.0001 2.8228 13.2748 <.0001 5.1668 10.2241 <.0001

II. Treatment comparisons of within-litter variances

i. E8 embryo

**Treatment** **Cr(III) vs Untreated Acid Saline vs Untreated Cr(III) vs Acid Saline**

**Covariate**   **p-value p-value p-value**

**Females**

**CpG19**  0.6283    0.0603   0.0290

**CpG20** 0.3131 0.0400 0.0043

**CpG21** 0.4144 0.0109 0.0019

**CpG22** 0.9265 0.0004 0.0010

**CpG23** 0.1302 <0.0001 0.0261

**T** <.0001 <.0001 0.8162

**ACC** 0.2073 0.0031 <.0001

**CGC** 0.0013 0.7773 0.0004

**CCA** 0.1628 <.0001 0.0048

**CCC** <.0001 <.0001 0.3235

**Males**

**CpG19** 0.3369 0.0278 0.0056

**CpG20** 0.0761 0.1462 0.0032

**CpG21** 0.7091 0.0348 0.0326

**CpG22** 0.0164 0.7970 0.0098

**CpG23** 0.7363 0.0307 0.0320

**T** <.0001 0.0068 <.0001

**ACC** 0.1668 0.5959 0.3695

**CGC** <.0001 0.5601 0.0007

**CCA** 0.0027 <.0001 0.0604

**CCC** 0.1133 0.0425 0.0008

ii. 6-week adult offspring lung

**Treatment** **Cr(III) vs Untreated Acid Saline vs Untreated Cr(III) vs Acid Saline**

**Covariate**   **p-value p-value p-value**

**Females**

**CpG19**  0.2462 0.6207 0.4820

**CpG20** 0.1720 0.4736 0.4873

**CpG21** 0.1308 0.2697 0.6471

**CpG22** 0.1575 0.5633 0.3775

**CpG23** 0.1130 0.2522 0.6220

**T**   0.0571 0.3433 0.0040

**ACC** 0.2752 0.1517 0.7657

**CGC** 0.5989 0.8291 0.7436

**CCA** 0.0541 0.5008 0.1884

**CCC** 0.8528 0.5796 0.7190

**Males**

**CpG19** 0.06328 0.02634 0.8035

**CpG20** 0.07127 0.01280 0.5752

**CpG21** 0.17198 0.01250 0.3117

**CpG22** 0.05085 0.00268 0.3662

**CpG23** 0.11009 0.00638 0.3180

**T** 0.6754 0.2580 0.1411

**ACC** 0.3973 0.0039 0.0572

**CGC** 0.0399 0.7748 0.0215

**CCA** 0.0785 0.1135 0.7943

**CCC** 0.0821 0.0103 0.4791

iii. 6-week adult offspring liver

**Treatment** **Cr(III) vs Untreated Acid Saline vs Untreated Cr(III) vs Acid Saline**

**Covariate**   **p-value p-value p-value**

**Females**

**CpG19**  0.1960     0.0226    0.3381

**CpG20** 0.1624 0.0185 0.3541

**CpG21** 0.2272 0.0141 0.2210

**CpG22** 0.0899 0.0073 0.3419

**CpG23** 0.2765 0.0267 0.2697

**T**  0.7137 0.1528 0.2944

**ACC** 0.8888 0.5533 0.6558

**CGC** 0.3180 0.0148 0.1608

**CCA** 0.7884 0.1697 0.2744

**CCC** 0.0010 0.3867 0.0111

**Males**

**CpG19** 0.8560 0.7666 0.9209

**CpG20** 0.5152 0.3384 0.7978

**CpG21** 0.8426 0.5978 0.7647

**CpG22** 0.4186 0.5402 0.8220

**CpG23** 0.2567 0.3914 0.7518

**T** 0.5709 0.0227 0.1136

**ACC** 0.4089 0.9592 0.3853

**CGC** 0.0169 0.9127 0.0136

**CCA** 0.8044 0.9283 0.7407

**CCC** 0.3712 0.3997 0.0938

iv. 6-week adult offspring sperm

**Treatment** **Cr(III) vs Untreated Acid Saline vs Untreated Cr(III) vs Acid Saline**

**Covariate**   **p-value p-value p-value**

**CpG19** 0.3864 0.5438 0.14392

**CpG20** 0.3042 0.8857 0.23284

**CpG21** 0.2214 0.9406 0.23417

**CpG22** 0.2985 0.5743 0.11056

**CpG23** 0.7638 0.1443 0.09164

**T** 0.0074 0.8485 0.0035

**ACC** 0.5855 0.8106 0.7368

**CGC** 0.0779 0.4162 0.0103

**CCA** 0.0668 0.7826 0.0325

**CCC** 0.1994 0.8747 0.2392
