# Supplementary material for: Ontogeny-Driven rDNA Rearrangement, Methylation, and Transcription, and Paternal Influence
Source: PLoS One. 2011 Jul 12;6(7):e22266. doi: 10.1371/journal.pone.0022266 (PMC3134480; doi:10.1371/journal.pone.0022266)
Supplement: Data S2 — Mixed-effects model of regression slopes between CpG methylation and sequence variants. (DOC) [file pone.0022266.s005.doc]

Data S2. Mixed-effects model of regression slopes between CpG methylation and sequence variants (red: p<0.05, with a negative association; yellow: p<0.05, with a positive association).

I. Sperm of male mice 2-week posttreatment

**Treatment Untreated Acid Saline Cr(III)** Sequence variant Estimate p-value Estimate p-value Estimate p-value

**CPG19**  **CPG19**  **CPG19**

**T**  -0.89693 0.00001 -0.88605 0.00026 -0.49829 0.08121

**ACC**  0.18836 0.18935 0.00516 0.97148 0.20122 0.16087

**CGC**  -0.99445 0.00000 -0.57842 0.00777 -0.66147 0.00002

**CCA**  -0.46556 0.02641 -0.11307 0.59315 0.09870 0.64883

**CCC**  1.03817 0.00000 0.64995 0.00154 0.56985 0.00999

**CPG20**  **CPG20**  **CPG20**

**T**  -0.82533 0.00000 -0.80618 0.00022 -0.41251 0.10815

**ACC**  0.21601 0.09238 0.02752 0.83227 0.18507 0.15209

**CGC**  -0.84418 0.00000 -0.54314 0.00587 -0.61012 0.00001

**CCA**  -0.51791 0.00507 -0.12524 0.50901 0.12096 0.53347

**CCC**  0.88407 0.00000 0.57352 0.00214 0.48991 0.01495

**CPG21**  **CPG21**  **CPG21**

**T**  -0.75514 0.00001 -0.63145 0.00266 -0.40548 0.10318

**ACC**  0.23757 0.05276 0.03705 0.76518 0.18526 0.13324

**CGC**  -0.83137 0.00000 -0.47039 0.01277 -0.56415 0.00002

**CCA**  -0.48982 0.00562 -0.11774 0.51662 0.09452 0.61117

**CCC**  0.80023 0.00001 0.47947 0.00784 0.43863 0.02423

**CPG22**  **CPG22**  **CPG22**

**T**  -0.86881 0.00001 -0.91243 0.00014 -0.49025 0.08162

**ACC**  0.22968 0.10255 -0.01169 0.93459 0.18392 0.19399

**CGC**  -1.05396 0.00000 -0.55381 0.00945 -0.65352 0.00001

**CCA**  -0.47203 0.01989 -0.11400 0.58465 0.16374 0.44352

**CCC**  1.00549 0.00000 0.66101 0.00117 0.52229 0.01711

**CPG23**  **CPG23**  **CPG23**

**T**  -0.99355 0.00000 -1.04627 0.00005 -0.49680 0.10035

**ACC**  0.32314 0.03134 0.01391 0.92690 0.29254 0.05286

**CGC**  -1.25799 0.00000 -0.53098 0.01847 -0.73676 0.00000

**CCA**  -0.57337 0.00845 -0.17975 0.42169 0.03949 0.86309

**CCC**  1.12793 0.00000 0.65835 0.00254 0.54582 0.02020

II. E8 embryo

**Treatment Untreated Acid Saline Cr(III)**  Sequence variant Estimate p-value Estimate p-value Estimate p-value

**Females**

**CPG19**  **CPG19**  **CPG19**

**T**  -0.41403 0.01401 -0.78312 0.00000 -0.40588 0.05326

**ACC** -0.17705 0.09558 -0.34060 0.00343 -0.11754 0.40261

**CGC** 0.06097 0.58018 -0.10610 0.31882 -0.29232 0.02202

**CCA** 0.01637 0.88185 -0.17472 0.18587 0.07242 0.68737

**CCC** 0.03392 0.63754 0.39774 0.00000 0.29422 0.00723

**CPG20**  **CPG20**  **CPG20**

**T**  -0.39529 0.00300 -0.80073 0.00000 -0.38601 0.01987

**ACC** -0.17357 0.08134 -0.36698 0.00085 -0.09793 0.46163

**CGC** 0.10451 0.26194 -0.19955 0.02619 -0.28610 0.01012

**CCA** -0.03973 0.69374 -0.21154 0.08534 0.11367 0.49154

**CCC** 0.03534 0.58229 0.42529 0.00000 0.26013 0.00932

**CPG21**  **CPG21**  **CPG20**

**T**  -0.37473 0.00424 -0.81528 0.00000 -0.44614 0.00688

**ACC** -0.17062 0.06439 -0.33890 0.00114 -0.10161 0.41802

**CGC** 0.10823 0.26896 -0.25155 0.00783 -0.33946 0.00306

**CCA** -0.03997 0.66829 -0.15488 0.19098 -0.04743 0.76493

**CCC** 0.03485 0.57986 0.45538 0.00000 0.36682 0.00024

**CPG22**  **CPG22**  **CPG20**

**T**  -0.31852 0.05979 -0.84427 0.00000 -0.42827 0.04155

**ACC** -0.25151 0.03738 -0.41937 0.00134 -0.15116 0.33743

**CGC** 0.17390 0.14717 -0.07917 0.49299 -0.32480 0.01943

**CCA** -0.00904 0.94320 -0.27392 0.06644 0.13406 0.51081

**CCC** 0.07740 0.37910 0.43315 0.00001 0.34471 0.00588

**CPG23**  **CPG23**  **CPG20**

**T**  -0.30546 0.09176 -0.95097 0.00000 -0.46604 0.03946

**ACC** -0.14709 0.24337 -0.43599 0.00165 0.03220 0.84561

**CGC** 0.03839 0.76231 -0.15837 0.19414 -0.46112 0.00191

**CCA** -0.06430 0.59077 -0.29723 0.05330 0.05372 0.79330

**CCC** 0.10866 0.24955 0.53836 0.00000 0.43956 0.00143

**Males**

**CPG19**  **CPG19**  **CPG19**

**T**  -0.48012 0.00028 -0.50297 0.00034 -0.55763 0.00105

**ACC**  -0.17864 0.06961 -0.33675 0.00233 -0.10799 0.44224

**CGC**  -0.06034 0.61392 -0.16686 0.14803 -0.07750 0.52390

**CCA**  -0.04613 0.70975 0.07133 0.63997 0.07028 0.74786

**CCC**  0.25587 0.00811 0.42025 0.00009 0.14454 0.21851

**CPG20**  **CPG20**  **CPG20**

**T**  -0.48109 0.00008 -0.55561 0.00002 -0.55937 0.00023

**ACC**  -0.11022 0.21751 -0.36019 0.00044 -0.07528 0.56261

**CGC**  -0.08356 0.43572 -0.13947 0.17960 -0.09972 0.35823

**CCA**  -0.02508 0.81809 0.08304 0.54813 -0.04630 0.81501

**CCC**  0.17081 0.05232 0.35501 0.00030 0.18993 0.07868

**CPG21**  **CPG21**  **CPG21**

**T**  -0.46770 0.00010 -0.60516 0.00000 -0.64381 0.00002

**ACC**  -0.12579 0.15987 -0.32673 0.00135 -0.03185 0.80380

**CGC**  -0.08091 0.44010 -0.14691 0.14951 -0.04695 0.64359

**CCA**  -0.05425 0.60808 0.00798 0.95327 -0.04873 0.80376

**CCC**  0.22608 0.01532 0.37750 0.00025 0.18287 0.10972

**CPG22**  **CPG22**  **CPG22**

**T**  -0.44276 0.00256 -0.63795 0.00004 -0.56329 0.00201

**ACC**  -0.14753 0.17474 -0.44064 0.00025 -0.12885 0.39982

**CGC**  -0.06798 0.57234 -0.05041 0.66693 -0.05881 0.62901

**CCA**  -0.00752 0.95623 -0.00385 0.98146 0.06008 0.79875

**CCC**  0.21029 0.05159 0.41555 0.00045 0.23974 0.07062

**CPG23**  **CPG23**  **CPG23**

**T**  -0.50900 0.00060 -0.64930 0.00010 -0.66760 0.00090

**ACC**  -0.12727 0.27634 -0.41167 0.00185 -0.10962 0.50398

**CGC**  -0.07814 0.56787 -0.11709 0.38143 0.05410 0.66521

**CCA**  -0.08361 0.56321 0.04513 0.80423 -0.02045 0.93804

**CCC**  0.25479 0.03897 0.47995 0.00043 0.19236 0.19553

III. 6-week adult offspring lung

**Treatment Untreated Acid Saline Cr(III)**  Sequence variant Estimate p-value Estimate p-value Estimate p-value

**Females**

**CPG19**  **CPG19**  **CPG19**

**T**  -0.35643 0.00490 -0.26175 0.022366 -0.63400 0.000001

**ACC**  -0.01275 0.87126 -0.33637 0.000042 -0.25200 0.004083

**CGC**  -0.45859 0.00002 -0.47261 0.000010 -0.42478 0.000082

**CCA**  -0.02885 0.75565 0.37940 0.000049 0.28658 0.007823

**CCC**  0.49400 0.00001 0.59833 0.000000 0.63927 0.000000

**CPG20**  **CPG20**  **CPG20**

**T**  -0.30330 0.00739 -0.26046 0.010784 -0.56980 0.000001

**ACC**  0.01411 0.85175 -0.31040 0.000072 -0.23148 0.005498

**CGC**  -0.41281 0.00006 -0.45003 0.000008 -0.35492 0.000541

**CCA**  -0.05965 0.49698 0.35356 0.000065 0.26652 0.008933

**CCC**  0.41485 0.00008 0.57672 0.000000 0.54372 0.000000

**CPG21**  **CPG21**  **CPG21**

**T**  -0.30346 0.00590 -0.23694 0.017394 -0.54596 0.000001

**ACC**  0.00267 0.97128 -0.31617 0.000039 -0.23766 0.003736

**CGC**  -0.40246 0.00005 -0.44608 0.000006 -0.35238 0.000390

**CCA**  -0.04756 0.57730 0.36037 0.000029 0.26917 0.006723

**CCC**  0.41536 0.00005 0.56962 0.000000 0.55110 0.000000

**CPG22**  **CPG22**  **CPG22**

**T**  -0.34513 0.00783 -0.29313 0.012176 -0.63489 0.000002

**ACC**  -0.06336 0.44610 -0.34106 0.000068 -0.29420 0.001258

**CGC**  -0.45763 0.00003 -0.51639 0.000001 -0.40783 0.000181

**CCA** 0.01494 0.87850 0.37400 0.000125 0.35071 0.001658

**CCC**  0.52314 0.00001 0.67106 0.000000 0.64645 0.000000

**CPG23**  **CPG23**  **CPG23**

**T**  -0.36551 0.01063 -0.29963 0.020919 -0.72142 0.000001

**ACC**  0.00063 0.99424 -0.36818 0.000059 -0.26770 0.006698

**CGC**  -0.50154 0.00005 -0.52836 0.000012 -0.46617 0.000148

**CCA**  -0.02820 0.78253 0.40749 0.000084 0.32798 0.006764

**CCC**  0.51018 0.00007 0.67479 0.000000 0.69924 0.000000

**Males**

**CPG19**  **CPG19**  **CPG19**

**T**  -0.39534 0.00072 -0.36678 0.00152 -0.51824 0.00013

**ACC**  -0.01657 0.85970 -0.24261 0.01742 -0.12680 0.22632

**CGC**  -0.65071 0.00000 -0.43530 0.00007 -0.43245 0.00071

**CCA**  0.03937 0.72877 0.19179 0.11678 0.04161 0.76674

**CCC**  0.63357 0.00000 0.61140 0.00000 0.54585 0.00002

**CPG20**  **CPG20**  **CPG20**

**T**  -0.38866 0.00044 -0.33054 0.00259 -0.48454 0.00017

**ACC** -0.00949 0.91681 -0.21094 0.03227 -0.11509 0.25604

**CGC**  -0.62300 0.00000 -0.39770 0.00018 -0.38189 0.00194

**CCA**  0.00150 0.98915 0.13206 0.26648 0.02195 0.87193

**CCC**  0.61336 0.00000 0.58689 0.00000 0.48747 0.00012

**CPG21**  **CPG21**  **CPG21**

**T**  -0.35759 0.00100 -0.32918 0.00229 -0.46346 0.00025

**ACC**  -0.00356 0.96740 -0.20031 0.03450 -0.11792 0.22586

**CGC**  -0.60579 0.00000 -0.40266 0.00010 -0.37725 0.00166

**CCA**  0.01030 0.92261 0.14114 0.21656 0.01968 0.88081

**CCC**  0.57033 0.00000 0.56983 0.00000 0.48699 0.00006

**CPG22**  **CPG22**  **CPG22**

**T**  -0.36370 0.00307 -0.37560 0.00198 -0.52729 0.00020

**ACC**  -0.00738 0.93896 -0.18257 0.08188 -0.14806 0.16961

**CGC**  -0.67612 0.00000 -0.43046 0.00018 -0.42336 0.00148

**CCA**  0.02697 0.82001 0.11787 0.35649 0.02201 0.87995

**CCC**  0.60966 0.00001 0.58442 0.00000 0.57031 0.00003

**CPG23**  **CPG23**  **CPG23**

**T**  -0.42431 0.00193 -0.42263 0.00186 -0.62700 0.00007

**ACC**  -0.00167 0.98722 -0.27583 0.01548 -0.14417 0.21681

**CGC**  -0.73345 0.00000 -0.50985 0.00002 -0.49884 0.00041

**CCA**  0.02651 0.83535 0.22847 0.09702 0.03426 0.82775

**CCC**  0.65539 0.00001 0.70368 0.00000 0.63433 0.00001

IV. 6-week adult offspring liver

**Treatment Untreated Acid Saline Cr(III)**  Sequence variant Estimate p-value Estimate p-value Estimate p-value

**Females**

**CPG19**  **CPG19**  **CPG19**

**T**  -0.31605 0.00115 -0.22632 0.01045 -0.22955 0.01586

**ACC** -0.03226 0.64647 -0.39910 0.00000 -0.16468 0.03878

**CGC** -0.42130 0.00002 -0.44830 0.00008 -0.14229 0.17468

**CCA** -0.03330 0.77894 0.43406 0.00002 0.14378 0.19495

**CCC** 0.38960 0.00058 0.69804 0.00000 0.41557 0.00124

**CPG20**  **CPG20**  **CPG20**

**T**  -0.31318 0.00035 -0.19926 0.01219 -0.22460 0.00874

**ACC** -0.01790 0.78637 -0.32884 0.00000 -0.13412 0.07575

**CGC** -0.37939 0.00003 -0.39394 0.00014 -0.14096 0.14004

**CCA** -0.06375 0.55407 0.37066 0.00008 0.11417 0.26971

**CCC** 0.35166 0.00065 0.57779 0.00000 0.34004 0.00369

**CPG21**  **CPG21**  **CPG21**

**T**  -0.29784 0.00046 -0.19695 0.01082 -0.21202 0.01097

**ACC** -0.00949 0.88220 -0.32121 0.00000 -0.13660 0.06230

**CGC** -0.35873 0.00003 -0.40070 0.00005 -0.13343 0.14434

**CCA** -0.06132 0.56243 0.35984 0.00010 0.12209 0.22758

**CCC** 0.31492 0.00197 0.57883 0.00000 0.33692 0.00345

**CPG22**  **CPG22**  **CPG22**

**T**  -0.28113 0.00338 -0.20104 0.0216 -0.24254 0.009817

**ACC** -0.02848 0.68252 -0.38951 0.0000 -0.14255 0.070940

**CGC** -0.40950 0.00001 -0.45360 0.0000 -0.15083 0.131633

**CCA** -0.02349 0.84098 0.43833 0.0000 0.12404 0.253981

**CCC** 0.37264 0.00070 0.68411 0.0000 0.39096 0.001760

**CPG23**  **CPG23**  **CPG23**

**T**  -0.29954 0.00813 -0.21736 0.0336 -0.23321 0.036622

**ACC** -0.01578 0.84382 -0.41269 0.0000 -0.17659 0.047900

**CGC** -0.45201 0.00002 -0.51018 0.0000 -0.13320 0.238073

**CCA** -0.03758 0.77614 0.44388 0.0000 0.14102 0.251027

**CCC** 0.37113 0.00299 0.75807 0.0000 0.46061 0.001220

**Males**

**CPG19**  **CPG19**  **CPG19**

**T**  -0.33365 0.00062 -0.12701 0.17441 -0.12313 0.30912

**ACC**  0.02887 0.74112 -0.24176 0.00946 -0.02903 0.77027

**CGC**  -0.42653 0.00004 -0.38185 0.00102 -0.45114 0.00093

**CCA**  -0.02551 0.82525 0.26956 0.02293 0.06595 0.62983

**CCC**  0.45302 0.00009 0.41821 0.00133 0.56699 0.00011

**CPG20**  **CPG20**  **CPG20**

**T**  -0.34865 0.00008 -0.13359 0.11600 -0.15953 0.14837

**ACC**  0.05737 0.48704 -0.19465 0.02564 -0.05252 0.57361

**CGC**  -0.37081 0.00005 -0.30862 0.00267 -0.42290 0.00049

**CCA**  -0.11336 0.28452 0.19419 0.07342 0.08555 0.49466

**CCC**  0.40893 0.00004 0.33244 0.00359 0.54671 0.00002

**CPG21**  **CPG21**  **CPG21**

**T**  -0.35538 0.00004 -0.13866 0.09258 -0.16295 0.12959

**ACC**  0.03831 0.63175 -0.20800 0.01392 -0.06021 0.50560

**CGC**  -0.36859 0.00003 -0.31450 0.00155 -0.40572 0.00057

**CCA**  -0.09751 0.33852 0.21395 0.04049 0.08462 0.48370

**CCC**  0.42992 0.00001 0.34473 0.00193 0.55266 0.00001

**CPG22**  **CPG22**  **CPG22**

**T**  -0.36492 0.00009 -0.13537 0.12589 -0.11351 0.33190

**ACC**  0.05883 0.49551 -0.24317 0.00798 -0.05301 0.58756

**CGC**  -0.42981 0.00002 -0.39534 0.00039 -0.46217 0.00042

**CCA**  -0.07876 0.47690 0.28091 0.01334 0.08854 0.50007

**CCC**  0.44776 0.00004 0.42393 0.00055 0.59492 0.00002

**CPG23**  **CPG23**  **CPG23**

**T**  -0.35024 0.00114 -0.07979 0.43367 -0.12093 0.37301

**ACC**  0.06786 0.48653 -0.25698 0.01336 -0.02677 0.80862

**CGC**  -0.50415 0.00001 -0.39676 0.00166 -0.49452 0.00098

**CCA**  -0.08761 0.49021 0.28154 0.03037 0.03878 0.79691

**CCC**  0.51759 0.00002 0.40275 0.00374 0.65773 0.00003

V. 6-week adult offspring sperm

**Treatment Untreated Acid Saline Cr(III)**  Sequence variant Estimate p-value Estimate p-value Estimate p-value

**CPG19**  **CPG19**  **CPG19**

**T**  -0.27775 0.04005 -0.33311 0.010546 -0.77732 0.00012

**ACC** -0.09304 0.44739 -0.29318 0.014159 -0.06085 0.66066

**CGC**  -0.75040 0.00000 -0.42948 0.000029 -0.35958 0.01055

**CCA** 0.22340 0.10544 0.30829 0.016438 -0.15856 0.36838

**CCC**  0.67414 0.00000 0.52253 0.000005 0.42660 0.00213

**CPG20**  **CPG20**  **CPG20**

**T**  -0.27333 0.03706 -0.35330 0.005571 -0.69428 0.00031

**ACC** -0.12248 0.30086 -0.27509 0.018069 -0.06142 0.64609

**CGC**  -0.65596 0.00000 -0.35637 0.000246 -0.33910 0.01125

**CCA**  0.22461 0.08865 0.23108 0.060536 -0.12296 0.46347

**CCC**  0.65745 0.00000 0.46782 0.000014 0.38070 0.00361

**CPG21**  **CPG21**  **CPG21**

**T**  -0.28628 0.02483 -0.29284 0.019396 -0.66197 0.00047

**ACC**  -0.13191 0.25452 -0.27349 0.016841 -0.07180 0.58510

**CGC**  -0.59734 0.00000 -0.33834 0.000479 -0.33424 0.01233

**CCA**  0.20560 0.10247 0.24871 0.033767 -0.11611 0.47415

**CCC**  0.65241 0.00000 0.43189 0.000049 0.37268 0.00395

**CPG22**  **CPG22**  **CPG22**

**T**  -0.30246 0.02410 -0.33815 0.009182 -0.76896 0.00014

**ACC**  -0.16035 0.20011 -0.28541 0.018703 -0.05821 0.67883

**CGC**  -0.70415 0.00000 -0.39668 0.000085 -0.34252 0.01452

**CCA**  0.24790 0.07555 0.27309 0.035556 -0.15468 0.38409

**CCC**  0.71623 0.00000 0.49234 0.000019 0.41921 0.00280

**CPG23**  **CPG23**  **CPG23**

**T**  -0.31756 0.02586 -0.28764 0.036532 -0.81363 0.00014

**ACC**  -0.06469 0.61963 -0.30999 0.015235 -0.06941 0.63836

**CGC**  -0.71048 0.00000 -0.46218 0.000044 -0.38208 0.01241

**CCA**  0.15467 0.28466 0.32226 0.017220 -0.17595 0.34354

**CCC**  0.68048 0.00000 0.54158 0.000007 0.45650 0.00188
