# Supplementary material for: Ontogeny-Driven rDNA Rearrangement, Methylation, and Transcription, and Paternal Influence
Source: PLoS One. 2011 Jul 12;6(7):e22266. doi: 10.1371/journal.pone.0022266 (PMC3134480; doi:10.1371/journal.pone.0022266)
Supplement: Data S3 — Paired T test of tissue versus tissue and mixed-effects model of pairwise comparisons of treatments. (DOC) [file pone.0022266.s006.doc]

Data S3. Paired T test of tissue versus tissue and mixed-effects model of pairwise comparisons of treatments. (red: p<0.05, with a negative difference; yellow: p<0.05, with a positive difference).

### I. Mean difference between two tissues and paired T test for the difference

**Treatment** **Untreated Acid Saline Cr(III)**

**Estimate p-value Estimate p-value Estimate p-value**

**Females**

**Lung vs Liver**

**CPG19**  2.7089 0.0002 3.0092 <.0001 6.1452 <.0001

**CPG20**  2.5224 <.0001 2.9676 <.0001 5.4854 <.0001

**CPG21**  2.6311 <.0001 2.9806 <.0001 5.2752 <.0001

**CPG22**  3.1213 <.0001 3.6607 <.0001 6.4926 <.0001

**CPG23**  3.715 <.0001 4.1035 <.0001 6.5963 <.0001

**T**  -0.796 0.0103 -0.039 0.8720 -0.839 0.0030

**ACC**  -0.927 0.0660 -0.557 0.0835 -0.187 0.4807

**CGC**  1.31 0.0008 -0.444 0.1282 -1.006 0.0026

**CCA**  0.0745 0.8120 0.0835 0.6901 0.2516 0.1444

**CCC**  -0.539 0.2184 0.8486 0.0125 0.9274 0.0086

**Males**

**Lung vs Liver**

**CPG19**  2.158 0.0041 2.6366 <.0001 4.2811 <.0001

**CPG20**  2.2565 0.0013 2.6025 <.0001 4.1317 <.0001

**CPG21**  2.219 0.0013 2.5788 <.0001 4.1422 <.0001

**CPG22**  2.6297 0.0010 2.8771 <.0001 4.8912 <.0001

**CPG23**  3.1109 0.0002 3.7268 <.0001 4.2447 <.0001

**T**  -0.441 0.1125 0.176 0.4072 -1.117 <.0001

**ACC**  0.092 0.8811 0.586 0.0204 -0.013 0.9668

**CGC**  -0.208 0.6407 -0.666 0.0459 -0.817 0.0102

**CCA**  0.207 0.6021 -0.418 0.0619 0.105 0.6547

**CCC**  -0.048 0.9087 0.481 0.1592 0.703 0.0320

**Lung vs Sperm**

**CPG19**  -6.3966 <.0001 -3.7891 <.0001 -2.7304 0.0002

**CPG20**  -4.3966 <.0001 -2.0554 <.0001 -2.0832 0.0010

**CPG21**  -3.1274 0.0008 -0.9139 0.0157 -0.8023 0.2009

**CPG22**  -3.6801 0.0009 -1.4419 0.0019 -0.1671 0.8252

**CPG23**  -2.7471 0.0131 -0.2047 0.6919 0.513 0.5549

**T**  0.310 0.3106 1.088 <.0001 0.071 0.8298

**ACC**  0.902 0.1553 2.675 <.0001 1.839 <.0001

**CGC**  2.131 <.0001 -1.421 0.0033 0.755 0.1612

**CCA**  -0.346 0.3749 -1.732 <.0001 -1.705 <.0001

**CCC**  -2.694 <.0001 0.430 0.4018 -0.912 0.1565

**Liver vs Sperm**

**CPG19**  -6.412 <.0001 -5.6906 <.0001 -6.4185 <.0001

**CPG20**  -4.7794 <.0001 -4.1638 <.0001 -5.5769 <.0001

**CPG21**  -3.423 <.0001 -3.0894 <.0001 -4.5331 <.0001

**CPG22**  -4.3803 <.0001 -3.6758 <.0001 -4.43 <.0001

**CPG23**  -3.4554 <.0001 -3.0839 <.0001 -2.4179 0.0199

**T**  0.368 0.1802 0.452 0.0061 0.280 0.4662

**ACC**  0.561 0.0577 1.588 <.0001 2.069 <.0001

**CGC**  2.499 <.0001 -0.282 0.5601 0.694 0.1883

**CCA**  -1.668 <.0001 -1.629 <.0001 -1.799 <.0001

**CCC**  -1.492 0.0128 0.311 0.5822 -0.955 0.1144

II. Pairwise treatment comparisons in a mixed-effects model analysis

**Treatment** **Cr(III)-Untreated Acid Saline-Untreated Cr(III)-Acid Saline**

**Estimate p-value Estimate p-value Estimate p-value**

**Females**

**Lung vs Liver**

**CPG19**  3.201 0.0097 0.051 0.9657 3.150 0.0095

**CPG20**  2.801 0.0111 0.151 0.8874 2.650 0.0142

**CPG21**  2.467 0.0203 0.048 0.9625 2.419 0.0203

**CPG22**  3.292 0.0090 0.196 0.8721 3.097 0.0123

**CPG23**  2.684 0.0821 -0.002 0.9988 2.686 0.0766

**T**  -0.030 0.9595 0.848 0.1419 -0.878 0.1336

**ACC** 0.343 0.6584 0.285 0.7045 0.057 0.9399

**CGC** -2.223 0.0011 -1.586 0.0156 -0.637 0.3369

**CCA** 0.189 0.6625 -0.019 0.9646 0.207 0.6257

**CCC** 1.688 0.0242 1.351 0.0625 0.337 0.6451

**Males**

**Lung vs Liver**

**CPG19**  2.440 0.0584 0.638 0.6115 1.802 0.1628

**CPG20**  2.012 0.0744 0.462 0.6730 1.550 0.1705

**CPG21**  2.072 0.0705 0.441 0.6928 1.631 0.1553

**CPG22**  2.391 0.0708 0.347 0.7879 2.044 0.1232

**CPG23**  1.548 0.3156  0.737 0.6251 0.811 0.5996

**T**  -0.769 0.1628 0.395 0.4668 -1.165 0.0353

**ACC** 0.136 0.8950 0.257 0.8012 -0.121 0.9068

**CGC** -0.440 0.5518 -0.124 0.8648 -0.317 0.6708

**CCA** -0.288 0.6254 -0.708 0.2208 0.420 0.4785

**CCC** 1.068 0.1290 0.545 0.4301 0.523 0.4602

**Lung vs Sperm**

**CPG19**  3.568 0.0101 2.426 0.0660 1.143 0.3956

**CPG20**  2.258 0.0521 2.277 0.0383 -0.018 0.9870

**CPG21**  2.311 0.0305 2.197 0.0281 0.113 0.9125

**CPG22**  3.193 0.0339 1.957 0.1724 1.236 0.3979

**CPG23**  3.016 0.0468 2.338 0.1040 0.678 0.6450

**T**  -0.227 0.7009 0.845 0.1390 -1.071 0.0640

**ACC** 1.026 0.2282 1.697 0.0371 -0.670 0.4199

**CGC** -1.705 0.0937 -3.423 0.0005 1.718 0.0838

**CCA** -1.549 0.0104 -1.387 0.0164 -0.162 0.7823

**CCC** 2.279 0.0234 3.015 0.0019 -0.736 0.4517

**Liver vs Sperm**

**CPG19**  0.417 0.7167 -0.538 0.6217 0.955 0.3935

**CPG20**  0.910 0.3056 -0.561 0.4983 1.471 0.0886

**CPG21**  1.223 0.1880 -0.312 0.7186 1.535 0.0891

**CPG22**  0.443 0.6839 -0.487 0.6366 0.930 0.3803

**CPG23**  -0.336 0.8082 -0.210 0.8737 -0.126 0.9256

**T**  -0.850 0.1432 -0.911 0.1039 0.060 0.9150

**ACC** -1.418 0.0205 -1.146 0.0512 -0.273 0.6466

**CGC** 2.532 0.0208 3.013 0.0045 -0.480 0.6514

**CCA** 0.097 0.8309 -0.020 0.9629 0.117 0.7914

**CCC** -0.771 0.5182 -1.636 0.1585 0.865 0.4578
